# Pattern of lymph node metastases in gastric cancer: a side-study of the multicenter LOGICA-trial

**DOI:** 10.1007/s10120-022-01329-2

**Published:** 2022-09-14

**Authors:** Cas de Jongh, Lianne Triemstra, Arjen van der Veen, Lodewijk A. A. Brosens, Misha D. P. Luyer, Jan H. M. B. Stoot, Jelle P. Ruurda, Richard van Hillegersberg, the LOGICA Study Group, Hylke J. F. Brenkman, Maarten F. J. Seesing, Grard A. P. Nieuwenhuijzen, Jeroen E. H. Ponten, Juul J. W. Tegels, Karel W. E. Hulsewe, Bas P. L. Wijnhoven, Sjoerd M. Lagarde, Wobbe O. de Steur, Henk H Hartgrink, Ewout A. Kouwenhoven, Marc J van Det, Eelco B Wassenaar, P. van Duijvendijk, Werner A. Draaisma, Ivo A. M. J. Broeders, Donald L. van der Peet, Suzanne S. Gisbertz

**Affiliations:** 1 Department of Surgery, University Medical Center (UMC) Utrecht, G04.228. 3508 GA Utrecht, The Netherlands; 2 Department of Surgery, Catharina Hospital Eindhoven, Eindhoven, The Netherlands; 3 Department of Surgery, Zuyderland Medical Center, Sittard, The Netherlands; 4 Department of Pathology, UMC Utrecht, Utrecht, The Netherlands

**Keywords:** Gastric cancer, Lymphadenectomy, Lymph node metastasis, Personalized medicine

## Abstract

**Background:**

The relation between gastric cancer characteristics and lymph node (LN) metastatic patterns is not fully clear, especially following neoadjuvant chemotherapy (NAC). This study analyzed nodal metastatic patterns.

**Methods:**

Individual LN stations were analyzed for all patients from the LOGICA-trial, a Dutch multicenter randomized trial comparing laparoscopic versus open D2-gastrectomy for gastric cancer. The pattern of metastases per LN station was related to tumor location, cT-stage, Lauren classification and NAC.

**Results:**

Between 2015–2018, 212 patients underwent D2-gastrectomy, of whom 158 (75%) received NAC. LN metastases were present in 120 patients (57%). Proximal tumors metastasized predominantly to proximal LN stations (no. 1, 2, 7 and 9; *p* < 0.05), and distal tumors to distal LN stations (no. 5, 6 and 8; OR > 1, *p* > 0.05). However, distal tumors also metastasized to proximal LN stations, and vice versa. Despite NAC, each LN station (no. 1–9, 11 and 12a) showed metastases, regardless of tumor location, cT-stage, histological subtype and NAC treatment, including station 12a for cT1N0-tumors. LN metastases were present more frequently in diffuse versus intestinal tumors (66% versus 52%; *p* = 0,048), but not for cT3–4- versus cT1–2-stage (59% versus 51%; *p* = 0.259). However, the pattern of LN metastases was similar for these subgroups.

**Conclusions:**

The extent of lymphadenectomy cannot be reduced after NAC for gastric cancer. Although the pattern of LN metastases is related to tumor location, all LN stations contained metastases regardless of tumor location, cT-stage (including cT1N0-tumors), histological subtype, or NAC treatment. Therefore, D2-lymphadenectomy should be routinely performed during gastrectomy in Western patients.

**Supplementary Information:**

The online version contains supplementary material available at 10.1007/s10120-022-01329-2.

## Introduction

**Fig. 1:**
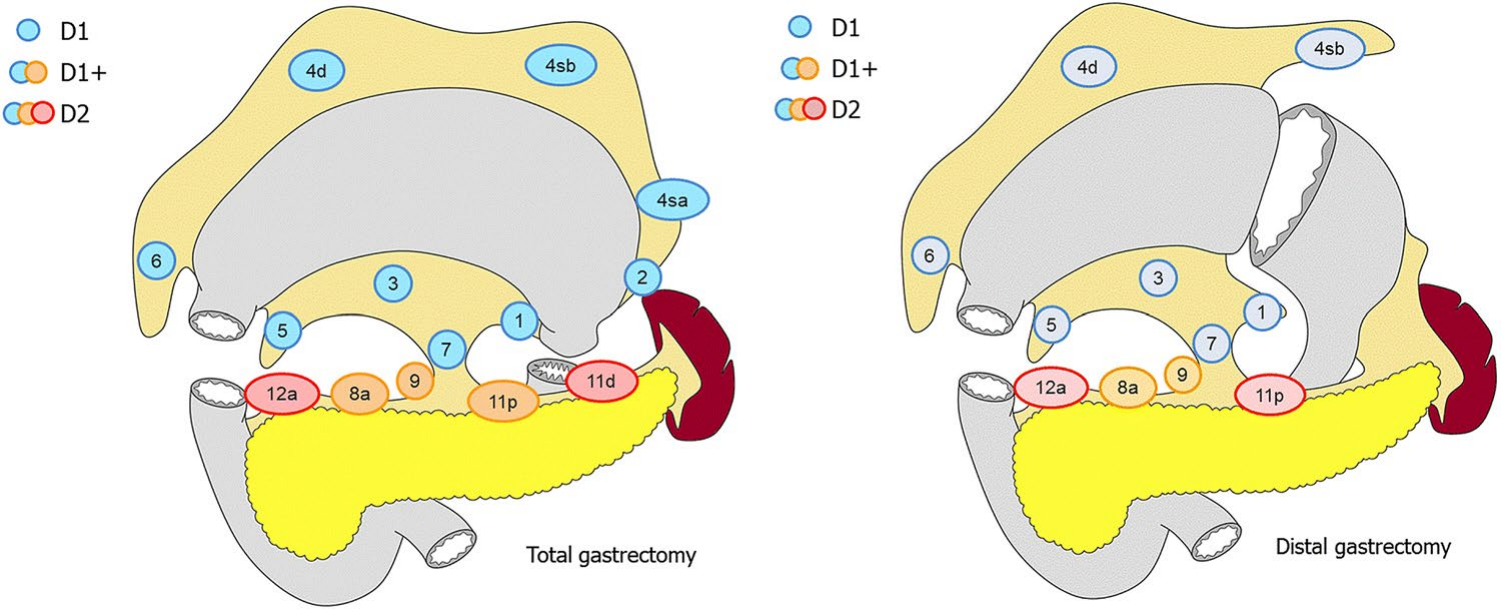
Lymph node stations for total and distal gastrectomy according to the 5th classification of the Japanese Gastric Cancer Association (JGCA) (10). The dissection of individual lymph node stations is displayed separately for total (left) and distal (right) gastrectomy, and also for D1- (blue stations), D1+ (blue and orange stations), and D2-lymphadenectomy. Of note: stations no. 13–20 and 110–112 are not depicted in this image. The original image was published by JGCA and can be found here: https://link.springer.com/article/10.1007/s10120-020-01042-y. No changes were made

Gastric cancer is the third leading cause of cancer deaths worldwide [1]. Gastrectomy with en-bloc lymphadenectomy combined with perioperative chemotherapy is the cornerstone of curative multimodality treatment for gastric cancer in most Western countries [2–7]. This results in a 36–45% 5-year survival. An adequate lymphadenectomy is of paramount importance as lymph node (LN) metastases are frequently present and negatively influence survival, and to adequately stage the disease and assess prognosis [5, 8, 9]. D2-lymphadenectomy is generally considered standard treatment for resectable gastric cancer [7, 10]. However, it has been suggested that the required extent of lymphadenectomy could vary per patient as the pattern of LN metastases may depend on tumor location and characteristics [11–20]. For instance, the Japanese Gastric Cancer Association (JGCA) recommends D1+-lymphadenectomy (without resecting stations 11 and 12a; Fig. 1) for cT1N0-tumors and advises different surgical lymphadenectomy strategies depending on tumor location [10].

Several studies investigated the pattern of LN metastases from gastric cancer following D2-/D3-lymphadenectomy [11–20]. Results showed that development of LN metastases is correlated with higher cT-stage and diffuse type tumors, and that location of locoregional LN metastases is related to primary tumor location [11–20]. However, these studies were retrospective and conducted decades ago, and mainly included Asian patients treated without neoadjuvant chemotherapy (NAC) [11–20]. Up to present day, neither prospective studies nor neoadjuvantly treated patients were investigated regarding this topic. Hence, this study’s aim was to assess the pattern of metastases per LN station in relation to tumor characteristics after D2-gastrectomy for gastric cancer in the multicenter randomized LOGICA-trial. The LOGICA-trial is ideally suited to investigate this aim due to the prospective study nature, standardized lymphadenectomy including separate collection of individual LN stations and prospective surgical quality control [21].

## Methods

### Study design

The LOGICA-trial (NCT02248519) evaluated surgical and oncological outcomes after randomization between a laparoscopic versus open approach for total and distal D2-gastrectomy for resectable gastric cancer [21]. Institutional review board approval was obtained at all ten Dutch participating centers and written informed consent was obtained for all patients.

### Patient selection

All LOGICA-patients were eligible for inclusion in this study. The inclusion and exclusion criteria were described in the LOGICA study protocol [21]. For this side-study, patients with D1-/D1+-lymphadenectomy or without resection of the primary tumor were excluded.

### Staging and treatment

The staging and perioperative chemotherapy treatment were determined in multidisciplinary tumor board meetings prior to treatment according to Dutch national guidelines, and were described in the LOGICA study protocol [7, 21]. Perioperative chemotherapy was recommended for all patients with advanced tumors (cT3–4- or cN+ -stage) who were deemed fit for this treatment.

Surgery included total or distal gastrectomy with en-bloc D2-lymphadenectomy combined with total omentectomy [21]. Distal gastrectomy was performed for antral and middle tumors, whereas tumors located in corpus and cardia and diffuse tumors were resected by total gastrectomy. D2-lymphadenectomy consisted of dissecting LN stations no. 1, 3, 4d + sb, 5–9, 11p and 12a for distal gastrectomy and LN stations 1–9, 11p/11d and 12a for total gastrectomy (Fig. 1).

### Surgical quality control

The previously published LOGICA study protocol describes the mandatory surgical quality control, consisting of central review of the performed lymphadenectomy by prospective assessment of intraoperative photographs, thereby providing active feedback after surgical procedures [21]. Furthermore, to ensure accurate results, the protocol mandated that all individual LN stations were collected in separate pathology containers (stations no. 8, 9, 11p, 11d and 12a) or were clearly marked at the resection specimen (all other stations). Additionally, surgeons divided the greater omentum in 4 quadrants (left/right and upper/lower) at the back-table in the operating room [21].

### Histopathological examination

The Dutch national guidelines were followed [7]. Pathologists described the status of all LN stations separately according to the JGCA-classification, also including regression after NAC per LN per station [10]. The original LOGICA-CRF was completed with details from pathology reports of all LOGICA-patients from all participating centers via PALGA, The Netherlands nationwide network and registry of histo-/cytopathology [22]. Stations 4sa, 4sb and 4d were grouped to station 4. Stations 11p and 11d were analyzed separately as no. 11d is resected for total gastrectomy, but not for distal gastrectomy. If pathology reports provided insufficient detail, local pathologists were contacted for clarification. If multiple LN stations were collected in the same pathology container, lymph nodes in that container were equally distributed over these stations. The Lauren histological intestinal and mixed type were grouped as intestinal tumors. Skip-metastases were defined as LN metastases located in remote, extra-perigastric stations only (no. 7–9, 11 and 12a), without involving the perigastric N1-stations (no. 1–6).

### Outcomes

The primary outcome was the pattern of metastases per individual LN station in relation to tumor location, clinical T-stage, Lauren classification (diffuse or intestinal type) and NAC treatment (yes/no). Clinical T-stage was used as surgical strategies are determined based on preoperative information. Proximal tumors were defined as tumors in cardia (Siewert type II/III according to the TNM-7-classification), fundus or upper one-third of the corpus; middle tumors as tumors in the remaining two-third of the corpus; and distal tumors as tumors in antrum or pylorus [10, 23]. Secondary, the incidence of regression to NAC both in LNs per individual station and the primary tumor (Mandard tumor regression grading) were assessed and related to Lauren classification [24, 25]. Furthermore, LNs and LN metastases per quadrant of the greater omentum were identified.

### Statistical analysis

Statistical analyses were performed using IBM SPSS Statistics version 27.0 (SPSS Inc. Chicago, USA). Quantitative values were expressed as medians with interquartile range (IQR) and categorical values as counts with percentages, calculated after excluding missing values. Data distributions were evaluated using boxplots and/or histograms. The incidence of metastases per LN station was descriptively reported for four subgroups based on tumor location, cT-stage, Lauren classification and NAC treatment, and several combinations of these subgroups. The incidence of LN metastases was related to the four subgroups using univariate and multivariate logistic regression, both overall (for all LN stations combined) and separately for each individual LN station. As sensitivity analysis, these logistic regression analyses were repeated for only the NAC-treated patients. Odds ratios (OR) were noted with 95% confidence intervals (CI). The association between Lauren classification and histopathological regression in the primary tumor and in LNs was assessed using *Χ*^*2*^-tests. A two-sided *p* < 0,05 was considered statistically significant for all tests.

## Results

### Patient characteristics

**Fig. 2:**
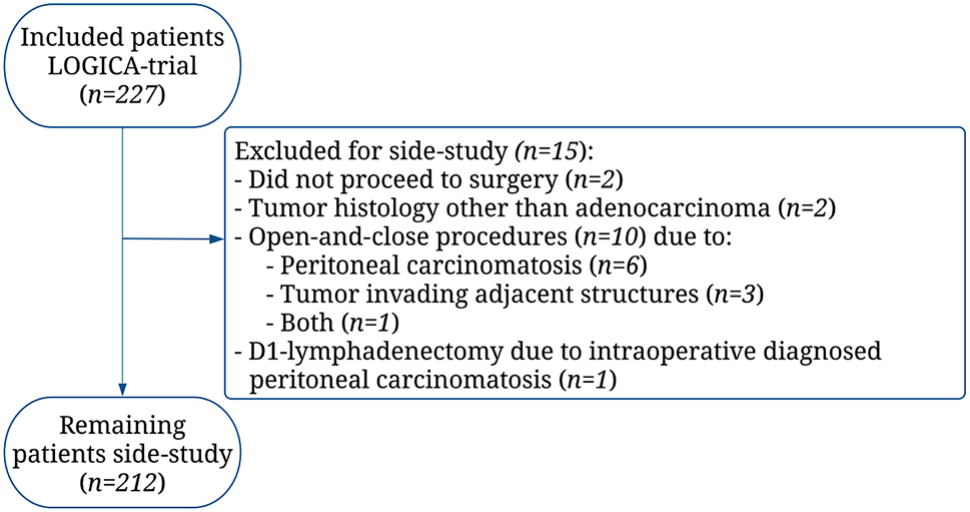
Study flowchart

Between February 2015 and August 2018, 212 of 227 LOGICA-patients (93%) were included in this study (Fig. 2). Reasons for exclusion (*n* = 15) were histology different from adenocarcinoma (*n* = 2), no resection of primary tumor due to medically inoperable patients (*n* = 2) or T4b-/M1-stages (*n* = 10), and D1-lymphadenectomy (*n* = 1) before diagnosing intraoperative peritoneal metastases.

Baseline and histopathological characteristics are summarized in Tables 1 and 2. Of the 212 patients, 120 (57%) were diagnosed with cT3-stage tumors. NAC was administered to 158 patients (75%), mostly the MAGIC-regimen or equivalent regimens (*n* = 120/158; 76%), FLOT-regimen (*n* = 29/158; 18%), or other regimens (*n* = 9; 6%). Total gastrectomy was performed in 90 patients (42%) and distal gastrectomy in 122 patients (58%). The median LN yield was 29 (IQR 21–39) per patient. In 120 patients (57%) LN metastases were detected, of whom 86 (72%) patients were treated with NAC.

**Table 1:**
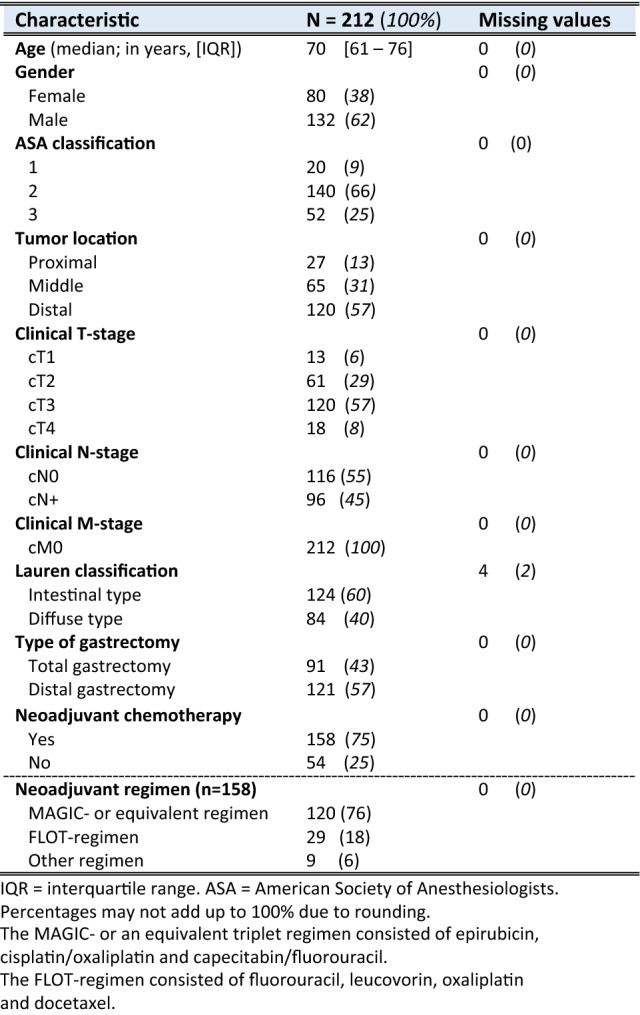
Baseline characteristics

**Table 2:**
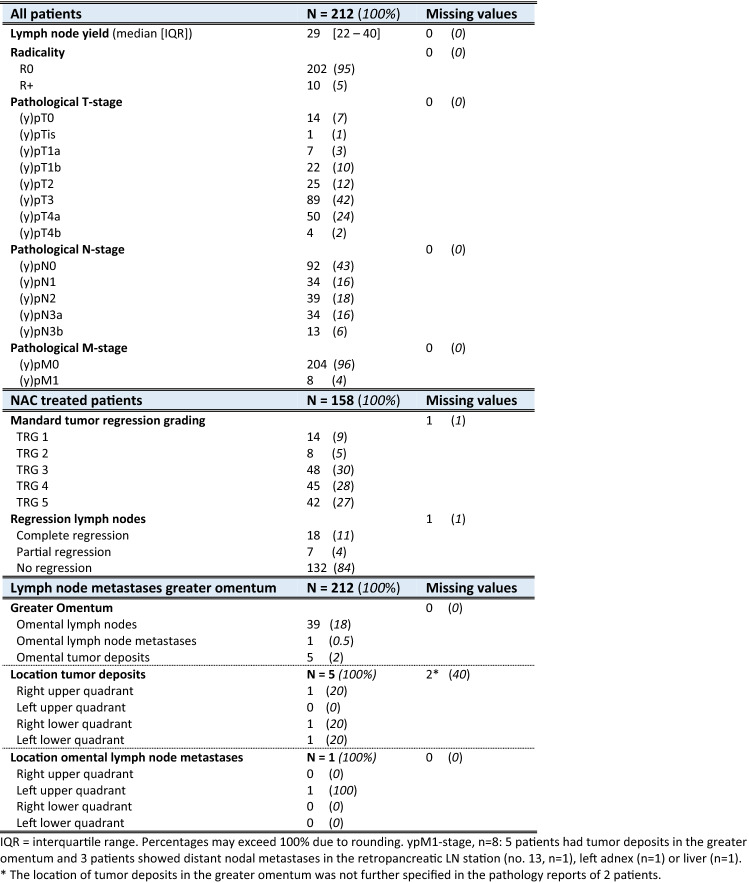
Histopathological results

### Distribution of lymph node metastases

**Fig. 3:**
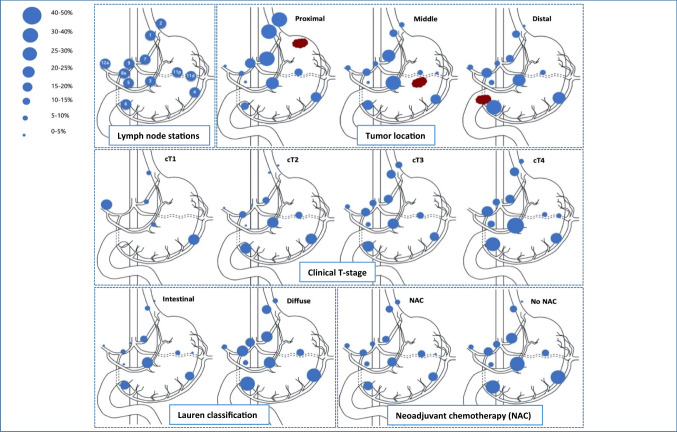
Incidence of lymph node metastases per tumor location, cT-stage, Lauren classification and treatment with or without neoadjuvant chemotherapy. The exact numbers for all incidences of lymph node metastases in this figure are displayed in Supplementary Table 1, which contains the same information, but numeric

Distribution of metastases per LN station is depicted for four patient subgroups based on tumor location, cT-stage, Lauren classification and NAC treatment (Fig. 3 and Supplementary Table 1), and for combinations of these subgroups (Table 3). LN metastases were detected in all individual resected stations (no. 1–9, 11, and 12a) for each tumor location (proximal, middle, and distal), for all cT-stages (cT1–4) and for both intestinal and diffuse tumors. LN stations 3 (23%), 4 (21%) and 6 (22%) were involved most frequently. Distal tumors were found in most cases (57%). For some patients (*n* = 16; 8%), LN stations were described grouped with one (*n* = 8) or multiple other stations (*n* = 8).

**Table 3:**
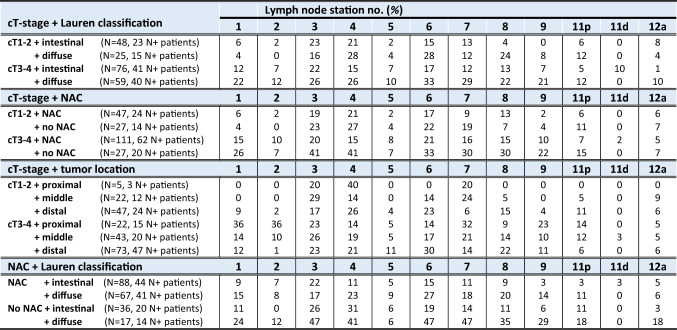
The incidence (%) of lymph node metastases per nodal station for subgroups

The subgroups are defined as (1) cT-stage + Lauren classification, (2) cT-stage + NAC, (3) Lauren classification + tumor location, and (4) Lauren classification + NAC

*cT*  clinical T-stage. *NAC*  neoadjuvant chemotherapy. Intestinal type = intestinal (*n* = 116) + mixed (*n* = 8) type. Station 11d was only resected during total gastrectomy

Percentages were calculated after excluding missing values

### Tumor location

Proximal tumors had more advanced tumor stages (cT3–4, 81%) than middle (66%) and distal tumors (61%). LN metastases were found most frequently for proximal tumors (63%), followed by distal (59%) and middle tumors (49%; *p* = 0,334). Overall (all LN stations analyzed combined, Table 4), tumor location did not predict developing LN metastases in multivariate analysis (*p* = 0,298). When assessing each LN station separately for all patients (Table 5), the tumor location was significantly related to location of LN metastases for proximal and distal tumors.

**Table 4:** Predictors of lymph node metastases (N0 versus N +) for the entire cohort (*n* = 212) and for only NAC-treated patients (*n* = 158), overall for all lymph node stations combined

Entire cohort						
*N* = 212, *n* = 120 N +	Univariable			Multivariable		
	OR	[95% CI]	*p*	OR	[95% CI]	*p*
Advanced stage (cT3-4)	1.39	[0.79–2.45]	0.259	1.32	[0.73–2.39]	0.353
Diffuse type	1.78	[1.00–3.15]	**0.048**	1.73	[0.97–3.09]	0.063
Location				0.334		0.298
Proximal	Ref.	–	–	Ref.	–	–
Middle	0.57	[0.23–1.43]	0.232	0.58	[0.23–1.49]	0.259
Distal	0.85	[0.36–2.02]	0.716	0.92	[0.38–2.23]	0.855

NAC-patients						
*N* = 158, *n* = 86 N +	Univariable			Multivariable		
	OR	[95% CI]	*p*	OR	[95% CI]	*p*
Advanced stage (cT3-4)	1.21	[0.61–2.40]	0.581	1.21	[0.60–2.44]	1.21
Diffuse type	1.58	[0.83–3.01]	0.166	1.55	[0.81–2.97]	1.55
Location			0.403			
Proximal	Ref.	–	–	Ref.	–	Ref
Middle	0.53	[0.19–1.45]	0.212	0.55	[0.20–1.53]	0.55
Distal	0.77	[0.31—1.94]	0.579	0.81	[0.31—2.09]	0.81

Bold indicates statistical significance (*p* < 0.05)

*NAC*  neoadjuvant chemotherapy. *OR*  odds ratio. 95% CI = 95% confidence interval

**Table 5:** Predictors of lymph node metastases (N0 versus N +) for the entire cohort (*n* = 212), for each lymph node station separately

LN station no. 1						
*N* = 212, 27 N + -patients	Univariable			Multivariable		
	OR	[95% CI]	*p*	OR	[95% CI]	*p*
Advanced stage (cT3-4)	3.48	[1.16–10.49]	**0.027**	2.81	[0.91–8.69]	0.074
Diffuse type	1.88	[0.82–4.29]	0.136	1.86	[0.79–4.40]	0.155
Location			**0.029**			0.050
Proximal	Ref.	–	–	Ref.	–	–
Middle	0.25	[0.08–0.81]	**0.021**	0.27	[0.08–0.89]	**0.031**
Distal	0.29	[0.11–0.79]	**0.016**	0.31	[0.11–0.88]	**0.028**

LN station no. 2						
*N* = 212, 14 N + -patients	Univariable			Multivariable		
	OR	[95% CI]	*p*	OR	[95% CI]	*p*
Advanced stage (cT3-4)	7.65	[0.98–9.71]	0.052	5.63	[0.69–45.96]	0.107
Diffuse type	1.81	[0.59–5.60]	0.302			
Location			** < 0.0005**			**0.001**
Proximal	Ref.	–	–	Ref.	–	–
Middle	0.16	[0.04–0.59]	**0.006**	0.18	[0.05–0.68]	**0.012**
Distal	0.04	[0.01–0.20]	** < 0.0005**	0.05	[0.01–0.25]	**0.000**

LN station no. 3						
*N* = 212, 48 N + -patients	Univariable			Multivariable		
	OR	[95% CI]	*p*	OR	[95% CI]	*p*
Advanced stage (cT3-4)	1.23	[0.62–2.45]	0.561	1.18	[0.58–2.38]	0.650
Diffuse type	1.01	[0.52–1.96]	0.983	1.00	[0.51–2.00]	0.991
Location			0.641			0.643
Proximal	Ref.	–	–	Ref.	–	–
Middle	1.29	[0.45–3.75]	0.636	1.33	[0.46–3.86]	0.606
Distal	0.92	[0.34–2.53]	0.873	0.94	[0.34–2.63]	0.913

LN station no. 4						
*N* = 212, 44 N + -patients	Univariable			Multivariable		
	OR	[95% CI]	*p*	OR	[95% CI]	*p*
Advanced stage (cT3-4)	0.81	[0.41–1.61]	0.544	0.76	[0.73–1.55]	0.448
Diffuse type	1.75	[0.89–3.45]	0.105	1.77	[0.90–3.51]	0.100
Location			0.617			0.717
Proximal	Ref.	–	–	Ref.	–	–
Middle	0.93	[0.29–3.00]	0.904	0.89	[0.27–2.89]	0.842
Distal	1.34	[0.46–3.86]	0.589	1.22	[0.41–3.61]	0.718

LN station no. 5						
*N* = 212, 13 N + -patients	Univariable			Multivariable		
	OR	[95% CI]	*p*	OR	[95% CI]	*p*
Advanced stage (cT3-4)	3.01	[0.67–14.38]	0.149	3.01	[0.67 – 14.38]	0.149
Diffuse type	1.80	[0.58–5.55]	0.309			
Location			0.354			
Proximal	Ref.	–	–			
Middle	0.85	[0.07–9.82]	0.898			
Distal	2.36	[0.29–19.29]	0.422			

LN station no. 6						
*N* = 212, 46 N + -patients	Univariable			Multivariable		
	OR	[95% CI]	*p*	OR	[95% CI]	*p*
Advanced stage (cT3-4)	1.31	[0.65–2.64]	0.457			
Diffuse type	2.37	[1.22–4.62]	**0.011**	2.33	[1.19–4.59]	**0.014**
Location			0.070			0.063
Proximal	Ref.	–	–	Ref.	–	–
Middle	1.48	[0.37–5.87]	0.576	1.49	[0.37–5.98]	0.576
Distal	3.03	[0.86–10.76]	0.086	3.14	[0.87–11.27]	0.080

LN station no. 7						
*N* = 212, 35 N + -patients	Univariable			Multivariable		
	OR	[95% CI]	*p*	OR	[95% CI]	*p*
Advanced stage (cT3-4)	1.67	[0.74–3.78]	0.222			
Diffuse type	2.29	[1.09–4.78]	**0.028**	2.49	[1.17–5.31]	**0.018**
Location			**0.027**			**0.025**
Proximal	Ref.	–	–	Ref.	–	–
Middle	0.68	[0.25–1.88]	0.455	0.66	[0.23–1.87]	0.435
Distal	0.29	[0.11–0.79]	**0.016**	0.27	[0.10–0.77]	**0.014**

LN station no. 8						
*N* = 212, 32 N + -patients	Univariable			Multivariable		
	OR	[95% CI]	*p*	OR	[95% CI]	*p*
Advanced stage (cT3-4)	1.75	[0.75–4.12]	0.199	1.79	[0.73–4.35]	0.202
Diffuse type	2.77	[1.26–6.08]	**0.011**	2.64	[1.19–5.85]	**0.017**
Location			0.175			0.164
Proximal	Ref.	–	–	Ref.	–	–
Middle	1.54	[0.30–7.92]	0.609	1.66	[0.32–8.77]	0.549
Distal	2.96	[0.66—13.42]	0.159	3.25	[0.70—15.22]	0.134

LN station no. 9						
*N* = 212, 19 N + -patients	Univariable			Multivariable		
	OR	[95% CI]	*p*	OR	[95% CI]	*p*
Advanced stage (cT3-4)	5.10	[1.15–22.72]	**0.033**	4.43	[0.96–20.43]	0.057
Diffuse type	4.83	[1.67–13.99]	**0.004**	4.77	[1.61– 14.16]	**0.005**
Location			0.183			0.261
Proximal	Ref.	–	–	Ref.	–	–
Middle	0.29	[0.07–1.19]	0.087	0.30	[0.07–1.31]	0.108
Distal	0.40	[0.13–1.29]	0.124	0.46	[0.13–1.61]	0.223

LN station no. 11						
*N* = 212, 18 N + -patients	Univariable			Multivariable		
	OR	[95% CI]	*p*	OR	[95% CI]	*p*
Advanced stage (cT3-4)	1.09	[0.39—3.03]	0.872			
Diffuse type	2.29	[0.84–6.28]	0.108	2.29	[0.84–6.28]	0.108
Location			0.799			
Proximal	Ref.	–	–			
Middle	0.83	[0.19–3.58]	0.800			
Distal	0.65	[0.16–2.58]	0.538			

LN station no. 12						
*N* = 212, 12 N + -patients	Univariable			Multivariable		
	OR	[95% CI]	*p*	OR	[95% CI]	*p*
Advanced stage (cT3-4)	0.74	[0.23–2.43]	0.623			
Diffuse type	2.19	[0.67–7.16]	0.194	2.19	[0.67–7.16]	0.194
Location			0.889			
Proximal	Ref.	–	–			
Middle	1.73	[0.19–16.27]	0.630			
Distal	1.61	[0.19–13.67]	0.662			

Bold indicates statistical significance (*p* < 0.05)

*NAC*  neoadjuvant chemotherapy. *OR*  odds ratio. 95% CI  95% confidence interval

Proximal tumors metastasized predominantly to proximal LN stations, most frequently to stations 1, 2, and 7. Compared to proximal tumors, significantly less metastases were found for distal tumors in nodal stations 1, 2, and 7 (no. 1 [OR 0.31, *p* = 0,028], no. 2 [OR 0.05, *p* < 0,0005], and no. 7 [OR 0.27, *p* = 0,014]) and for middle tumors in stations 1 and 2 (no. 1 [OR 0.27, *p* = 0,031] and no. 2 [OR 0.18, *p* = 0,012]). LN metastases from proximal tumors also involved distal stations (no. 5 and 6; 4% and 11%) and remote LN stations (no. 8, 11 and 12a; 7%, 11% and 4%).

Mid-gastric tumors metastasized most frequently to station 3, but metastases were more equally distributed over the different LN stations than proximal and distal tumors. LN metastases from mid-gastric tumors also involved all stations (no. 1–9, 11 and 12a), regardless of the cT-stage.

Distal tumors metastasized predominantly to distal LN stations, most frequently to stations 5, 6, and 8 (no. 5 [OR 2.36, *p* = 0,422], no. 6 [OR 3.14, *p* = 0,080], and no. 8 [OR 3.25, *p* = 0,134]). Distal tumors also involved proximal LN stations (no. 1, 2 and 7; 11%, 2%, and 11%) and remote LN stations (no. 8, 11, and 12a; 19%, 8%, and 6%), also for cT1–2-tumors.

### cT-stage

The highest incidence of LN metastases was found for cT4-tumors (72%) and cT3-tumors (58%). cT2-, cT3-, and cT4-stage tumors metastasized to all individual LN stations (no. 1–9, 11, and 12a). Of the 13 patients with cT1-stage, 11 patients (85%) had cN0-stage, but 4 patients (31%) showed histopathological LN metastases. Specifically, two patients (15%) with (distal) cT1N0-tumors showed metastases in remote station 12a. The distribution of LN metastases over the different stations was similar for cT1–2- versus cT3–4-tumors.

Overall (all LN stations analyzed combined), LN metastases were not significantly more often present for cT3–4- versus cT1–2-stage (59% versus 51%; OR 1.39, *p* = 0,259) in multivariate analysis (Table 4). When assessing each LN station separately for all patients (Table 5), cT3–4-tumors (versus cT1–2-stage) were a significant predictor for developing metastases in stations no. 1 (OR 3.48, *p* = 0,027) and 9 (OR 5.10, *p* = 0,033) in univariate analysis, but not in multivariate analysis.

In addition to clinical T-stage, pathological T-stage was assessed, showing similar distribution of LN metastases also after NAC (Supplementary Table 1). Stations 11 and 12a contained metastases for all (y)pT1–4-stages.

### Histological subtype

Diffuse tumors showed increased incidences of LN metastases versus intestinal tumors in almost all LN stations for both cT1–2-stage (60% versus 48%) and cT3–4-stage (68% versus 54%) and for both patients with (61% versus 50%) and without NAC (82% versus 56%), but the metastatic distribution of involved nodal stations was comparable to the intestinal type.

Overall (all LN stations analyzed combined), LN metastases were found significantly more often for diffuse versus intestinal tumors (66% versus 52%; OR 1.78, *p* = 0,048) in univariate analysis (Table 4), but not in multivariate analysis (OR 1.73 [0.97–3.09], *p* = 0,063). When assessing each LN station separately for all patients (Table 5), diffuse tumors revealed higher risk at LN metastases than intestinal tumors for all individual stations, with significantly increased OR in LN stations 6–9 (no. 6 [OR 2.33, *p* = 0,014], no. 7 [OR 2.49 *p* = 0,018], no. 8 [OR 2.64, *p* = 0,017], and no. 9 [OR 4.77, *p* = 0,005]).

### NAC treatment

LN metastases were detected more often in almost all stations for patients without NAC compared to patients treated with NAC (63% versus 54%; *p* = 0,275), especially for cT3–4-stage tumors (Table and Fig. 3). However, all LN stations (no. 1–9, 11 and 12a) showed metastases, and the distribution of LN metastases over the different stations was similar for patients with and without NAC.

The sensitivity analysis revealed a similar pattern of LN metastases in patients treated with NAC (n = 158/212, 75%) compared to the entire cohort (Supplementary Table 2).

### Skip-metastases

Fourteen patients (7%) demonstrated skip-metastases involving remote stations only (Supplementary Table 3). Stations 7 (3%) and 8 (4%) most frequently showed skip-metastases. A solitary skip-metastasis was found in station 7 (*n* = 4), 8 (*n* = 2), 11 (*n* = 1), and 12 (*n* = 1). Most of these 14 patients had cT3–4- and/or cN+-stages (*n* = 13, 93%) and distal tumors (*n* = 9, 64%).

### Histopathological response to NAC

A complete response in primary tumors was achieved in 14 out of 158 patients treated with NAC (9%), whereas 18 patients (11%) noted complete regression in LN metastases (Table 2). Compared to the entire cohort (*n* = 212), these 18 patients demonstrated similar metastatic incidences in LN stations 11 (12% versus 13%) and 12a (6% versus 6%). The histopathological response rates did not differ significantly for the intestinal versus diffuse type in primary tumors (*p* = 0,678) nor in lymph nodes (*p* = 0,449) (Supplementary Table 4). No regression at all was found in lymph nodes for 132 patients (84%) and the primary tumor for 42 patients (27%).

### Greater omentum

The greater omentum harbored LNs in 39 patients (18%), and only 1/212 patients (0.5%) showed LN metastases (Table 2). This patient had a distal cT3-tumor (diffuse type) with 4 LN metastases located in the upper left quadrant. Another 5 patients (2%) exhibited tumor deposits in the greater omentum, located in multiple quadrants. These 5 patients had cT3- (*n* = 4) and cT4-tumors (*n* = 1) of the intestinal (*n* = 2) and diffuse (*n* = 3) type, located in the proximal (*n* = 1), middle (*n* = 1) and distal (*n* = 3) stomach.

## Discussion

This is the first prospective multicenter study on the pattern of LN metastases in gastric cancer patients. These results show that the extent of lymphadenectomy cannot be reduced based on neoadjuvant treatment status. In addition, despite a relation between the pattern of LN metastases and primary tumor location, gastric cancer metastasized to each individual LN station (no. 1–9, 11 and 12a), regardless of tumor location, cT-stage, Lauren histological subtype and neoadjuvant chemotherapy, including station 12a for cT1N0-tumors.

The extent of lymphadenectomy during gastrectomy has been studied for many years. Long-term follow-up results of the randomized Dutch D1/D2-trial and Italian Gastric Cancer Study showed that performing D2-lymphadenectomy (without routine pancreatosplenectomy) leads to survival benefit compared to D1-lymphadenectomy, and is safe in terms of morbidity and mortality if performed by well-trained surgeons in high-volume centers for both early and advanced gastric cancer in Western patients (42–46% stage IA/IB) [5, 26]. Hence, according to the Dutch national guidelines and JGCA, en-bloc D2-lymphadenectomy is considered standard treatment, which was therefore performed for all LOGICA-patients [5, 7, 10, 26]. Our results show that the primary tumor location is significantly related to the location of LN metastases. These findings are in line with previous studies that retrospectively evaluated patterns of LN metastases, including several large cohorts with > 1000 patients [11, 12, 14, 16, 17, 19, 20]. However, proximal tumors still developed LN metastases in distal stations (no. 5 and 6), and distal tumors still metastasized to proximal stations (no. 1, 2, 7 and 9), for both early and advanced gastric cancer. Despite administering NAC, patients in the current cohort presented with LN metastases in all individual stations (no. 1–9, 11p/11d and 12a) independent from tumor site (proximal, middle, and distal), cT-stage, and histological subtype (and also without NAC). Interestingly, all after NAC, station 12a harbored LN metastases for (distal) cT1N0-tumors (*n* = 2, 15% of cT1N0-tumors) and station 11 showed a solitary (skip-)metastasis in 3 patients (2%). These findings contrast with JGCA-guidelines that recommend D1+-lymphadenectomy for distal cT1N0-tumors, thus without resecting stations 11(d) and 12a for this subgroup [10]. Although the impact on survival was not assessed in the present study, the current findings suggest that station 11 and 12a should be regarded as locoregional and should routinely be resected during gastrectomy, also after NAC and also for distal early gastric cancer. This is in line with multiple previous studies [12, 13, 16–19]. Therefore, we recommend that D2-lymphadenectomy should be routinely performed during gastrectomy for all Western gastric cancer patients, and irrespective of their neoadjuvant treatment status.

An important prognostic factor for gastric cancer patients is histopathological response to NAC [30]. Interestingly, the response to NAC was less in lymph nodes as opposed to the response in primary tumors. A total of 132 patients (84%) did not show any regression at all in lymph nodes, while 27% of patients had no regression (Mandard 5) in the primary tumor. This is consistent with a previous retrospective study [31]. We performed sensitivity analyses with only NAC-treated patients to test the robustness of our main conclusions, which showed lower incidences of metastases per nodal station but a similar pattern of LN metastases (which nodal stations were involved) after NAC for both early and advanced gastric cancer, for both intestinal and diffuse type tumors and for good responders. These findings suggest that the extent of lymphadenectomy cannot be reduced based on neoadjuvant treatment status.

In this study, diffuse type adenocarcinomas demonstrated significantly higher risk for developing LN metastases, as was shown previously [27–29]. However, despite higher incidences of LN metastases among diffuse versus intestinal tumors for both early (60% versus 48%) and advanced gastric cancer (68% versus 54%), and for both patients with (61% versus 50%) and without NAC (82% versus 56%), the pattern of LN metastases (which nodal stations were involved) was equivalent and included metastases in stations 11 and 12a for both subtypes, despite NAC treatment. Therefore, (at minimum) D2-lymphadenectomy seems necessary to achieve adequate oncological control regardless of histological subtype, also after NAC.

The added oncological value of performing omentectomy (partial or complete) has been an ongoing topic of debate as the survival benefit is not undisputed, although it was reported safe to perform [32–34]. In 212 patients undergoing complete omentectomy, we found omental LN metastases in only 1 patient (0.5%) and tumor deposits in 5 patients (2%), all with advanced gastric cancer (cT3–4- and/or cN+-stages). Conversely, 97.5% of patients underwent omentectomy without clear oncological benefit. These rates are comparable to previous studies [34, 35]. Although the added value of omentectomy in advanced gastric cancer seems limited based on a large retrospective study [36], ongoing prospective randomized trials (OMEGA-trial, TOP-G-trial and JCOG1711-trial) may provide more definitive conclusions about (long-term) oncological results [33, 34, 37].

Lymph node station 10 (splenic hilum) was not routinely dissected and could therefore not be assessed. Two retrospective studies found that station 10 may contain metastases in advanced (T3–4) cancers in the proximal/middle stomach [12, 16]. However, the 5th JGCA-classification recommends performing standard D2-lymphadenectomy without station 10 as solid evidence is lacking [10]. Prospective studies including survival assessment are warranted to clarify the role of station 10.

Although the LOGICA-trial was prospective and randomized, pathology reports were collected retrospectively to supplement sufficient detail. This is a limitation as for 16 patients (8%), not all individual LN stations were described separately, but some were grouped with one or multiple other LN stations. However, since most LN stations (*n* = 8, 4%) were closely related (i.e., no. 1 + 2 combined, or 7 + 9), we believe that this does not impact the study conclusions. Furthermore, the degree of LN regression was often noted in pathology reports when present but not when absent, and may be slightly underreported. Additionally, the nationwide standardized pathology protocol made it impossible to display incidences of metastases in sub-stations 4sa, 4sb and 4d [38]. Finally, the aspect of multiple testing was not corrected for in regression analyses and the number of covariates (multivariate analyses) was limited due to limited number events (= LN metastases per station). Strengths of this study are that this is the first prospective, multicenter trial that examined the pattern of LN metastases in Western gastric cancer patients. Moreover, collecting LN stations in separate pathology containers (no. 7–9, 11p/d and 12a) and clear markings on resection specimens (no. 1–6), and the prospective surgical quality control (feedback to local centers after central assessment of the lymphadenectomy via intraoperative photographs) were mandatory in the LOGICA-trial. This resulted in high-quality surgical data and accurate assessment of the nodal metastases pattern. Hence, the LOGICA-trial was ideally suited to investigate this study’s aim.

Future studies may focus on molecular subtyping of gastric cancer to potentially design individual tailored surgical treatment strategies [39]. For instance, the microsatellite instable subtype is associated with more N0-status, seems to show impaired response to chemotherapy, and may have better prognosis without NAC. In contrast, genomically stable tumors may benefit from NAC but metastasize more frequently and have worse prognosis, which might justify more aggressive (surgical) treatment approaches [40–42].

In conclusion, this was the first prospective multicenter study to assess the pattern of LN metastases of gastric cancer for Western patients. The results showed that the extent of lymphadenectomy cannot be reduced based on the neoadjuvant treatment status. In addition, although the pattern of LN metastases is related to tumor location in gastric cancer, metastatic spread occurred in all stations, regardless of tumor location, cT-stage (including cT1N0-tumors), histological subtype, or NAC treatment. Therefore, the results of the present study strongly support that D2-lymphadenectomy (including stations 11/12a) should be routinely performed during total and distal gastrectomy for gastric cancer in Western patients, also after administering neoadjuvant chemotherapy. Future research may focus on identifying other strategies to accomplish an individual tailored surgical treatment.

## Supplementary Information

Below is the link to the electronic supplementary material.

Supplementary file1 (DOCX 237 KB)
