# Supplementary material for: Pattern of lymph node metastases in gastric cancer: a side-study of the multicenter LOGICA-trial
Source: Gastric Cancer. 2022 Sep 14;25(6):1060–72. doi: 10.1007/s10120-022-01329-2 (PMC9587950; doi:10.1007/s10120-022-01329-2)
Supplement: Supplementary file 1 — Supplementary file1 (DOCX 237 KB) [file 10120_2022_1329_MOESM1_ESM.docx]

**Pattern of lymph node metastases in gastric cancer:**

**a side-study of the multicenter LOGICA-trial**

**AUTHORS:** Cas de Jongh MD^1^, Lianne Triemstra MD^1^, Arjen van der Veen MD^1^, Lodewijk AA Brosens MD PhD^1*^, Misha DP Luyer MD PhD^2^, Jan HMB Stoot MD PhD^3^, Jelle P Ruurda MD PhD^1^, Richard van Hillegersberg MD PhD^1^, on behalf of the LOGICA Study Group.

**COLLABORATORS (LOGICA Study Group):** Hylke JF Brenkman^1^, Maarten F.J. Seesing^1^, Grard AP Nieuwenhuijzen^2^, Jeroen EH Ponten^2^, Juul JW Tegels^3^, Karel WE Hulsewe^3^, Bas PL Wijnhoven^4^, Sjoerd M Lagarde^4^, Wobbe O de Steur^5^, Henk H Hartgrink^5^, Ewout A Kouwenhoven^6^, Marc J van Det^6^, Eelco B Wassenaar^7^, P. van Duijvendijk^7^, Werner A Draaisma^8^, Ivo AMJ Broeders^8^, Donald L van der Peet^9^, Suzanne S Gisbertz^10^.

**INSTITUTIONS AND AFFILIATIONS:** ^1^University Medical Center (UMC) Utrecht, Department of Surgery, Utrecht, The Netherlands. ^1*^UMC Utrecht, Department of Pathology, Utrecht, The Netherlands. ^2^Catharina Hospital Eindhoven, Department of Surgery, Eindhoven, The Netherlands. ^3^Zuyderland Medical Center, Department of Surgery, Sittard, The Netherlands. ^4^Erasmus UMC, Department of Surgery, Rotterdam, The Netherlands. ^5^Leiden UMC, Department of Surgery, Leiden, The Netherlands. ^6^ZGT Almelo, Department of Surgery, Almelo, The Netherlands. ^7^Gelre Hospitals Apeldoorn, Department of Surgery, Apeldoorn, The Netherlands. ^8^Meander Medical Center, Department of Surgery, Amersfoort, The Netherlands. ^9^Amsterdam UMC, Department of Surgery, University of Amsterdam, Amsterdam, The Netherlands. ^10^Amsterdam UMC, Department of Surgery, VU Amsterdam, Amsterdam, The Netherlands.

**CORRESPONDING AUTHOR CONCERNING THE MANUSCRIPT AND FOR REPRINTS:** Prof. dr. R. van Hillegersberg. Email: [R.vanHillegersberg@umcutrecht.nl](mailto:R.vanHillegersberg@umcutrecht.nl). UMC Utrecht, Department of Surgery, G04.228. 3508 GA, Utrecht, The Netherlands. T: +31 (0)88 - 755 8074. F: +31 (0)30 - 254 1944.

**MINI-ABSTRACT:** Although the nodal metastases pattern relate to tumor location, gastric cancer metastases occurred in all stations regardless of location, cT-stage, histological subtype or neoadjuvant chemotherapy. D2-lymphadenectomy should be routinely performed.

**Original article. Word count manuscript**: 3498. **Figures**: 3. **Tables**: 4. **Supplementary files**: 4.

**SUPPLEMENTARY MATERIAL**

***Supplementary Table 1.* Incidence of lymph node metastases per nodal station (%) for all patients (n=212), stratified in subgroups for neoadjuvant chemotherapy, cT-stage, Lauren classification, tumor location and (y)pT-stage.**

| **All patients and NAC** |  | **Lymph node station no. (*%*)** | | | | | | | | | | | | |
| --- | --- | --- | --- | --- | --- | --- | --- | --- | --- | --- | --- | --- | --- | --- |
|  | **1** | | **2** | **3** | **4** | **5** | **6** | **7** | **8** | **9** | **11p** | **11d^#^** | **12a** |  |
| **All patients**  (N=212, 120 N+ patients)  **NAC: yes** (N=158, 86 N+ patients)  **NAC: No** (N=54, 34 N+ patients) | 13  12  15 | | 7  8  4 | 23  20  32 | 21  17  34 | 6  6  6 | 22  20  28 | 17  14  25 | 15  14  19 | 9  8  13 | 13  12  13 | 1  1  0 | 6  5  7 |  |
|  |  | **Lymph node station no. (*%*)** | | | | | | | | | | | | |
| **cT-stage** | **1** | | **2** | **3** | **4** | **5** | **6** | **7** | **8** | **9** | **11p** | **11d^#^** | **12a** |  |
| **cT1**  (N=13, 4 N+ patients)  **cT2** (N=61, 34 N+ patients)  **cT3** (N=120, 68 N+ patients)  **cT4** (N=18, 13 N+ patients) | 8  5  17  17 | | 0  2  10  6 | 8  23  21  44 | 23  23  19  22 | 0  3  8  11 | 0  23  22  33 | 8  13  19  17 | 0  13  17  22 | 0  3  13  11 | 0  10  10  6 | 0  0  0  9 | 15*  3  6  0 |  |
|  |  | **Lymph node station no. (*%*)** | | | | | | | | | | | | |
| **Lauren classification** | **1** | | **2** | **3** | **4** | **5** | **6** | **7** | **8** | **9** | **11p** | **11d^#^** | **12a** |  |
| **Intestinal** (N=124, 64 N+ patients) **Diffuse** (N=84, 55 N+ patients) | 10  17 | | 5  8 | 23  23 | 17  27 | 5  8 | 16  31 | 12  24 | 10  23 | 4  17 | 6  12 | 2  0 | 4  8 |  |
|  |  | **Lymph node station no. (*%*)** | | | | | | | | | | | | |
| **Tumor location** | **1** | | **2** | **3** | **4** | **5** | **6** | **7** | **8** | **9** | **11p** | **11d^#^** | **12a** |  |
| **Proximal** (N=27, 17 N+ patients)  **Middle** (N=65, 32 N+ patients)  **Distal**  (N=120, 71 N+ patients) | 30  10  11 | | 30  6  2 | 22  27  21 | 19  18  23 | 4  3  8 | 11  16  28 | 30  22  11 | 7  11  19 | 19  6  8 | 11  9  8 | 0  3  0 | 4  6  6 |  |
|  | **Lymph node station no. (*%*)** | | | | | | | | | | | | |  |
| **(y)pT-stage** | **1** | | **2** | **3** | **4** | **5** | **6** | **7** | **8** | **9** | **11p** | **11d^#^** | **12a** |  |
| **(y)pT0** (N=14, 1 N+ patients)  **(y)pT1a/1b** (N=30, 11 N+ patients)  **(y)pT2** (N=25, 8 N+ patients)  **(y)pT3** (N=89, 62 N+ patients)  **(y)pT4a** (N=50, 34 N+ patients)  **(y)pT4b** (N=4, 4 N+ patients) | 0  0  4  16  24  50 | | 0  0  0  11  10  25 | 7  7  8  25  38  50 | 0  7  12  22  38  0 | 0  3  0  10  6  0 | 7  13  16  20  36  25 | 0  3  4  22  26  25 | 0  0  12  19  24  0 | 7  0  0  16  8  0 | 0  0  8  8  18  0 | 0  0  0  0  0  0 | 0  7  4  8  4  0 |  |

cT-stage = clinical T-stage. NAC = neoadjuvant chemotherapy. (y)pT-stage = pathological T-stage.

Please note that patients with (y)pT-stage can be after neoadjuvant chemotherapy, which can influence the incidence of nodal metastases.

# Station 11d was only resected during total gastrectomy.
***** 2 Patients with cT1N0-stage distal tumors showed LN metastases in station 12a.

The numbers in this table are displayed in a graphical way in Figure 3.


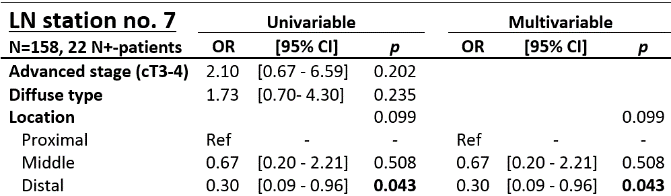

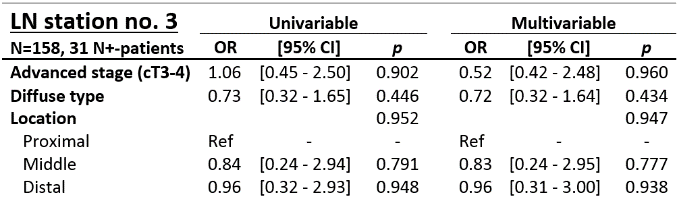

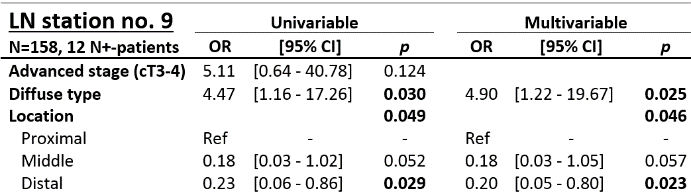

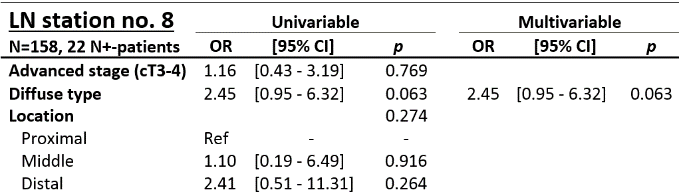

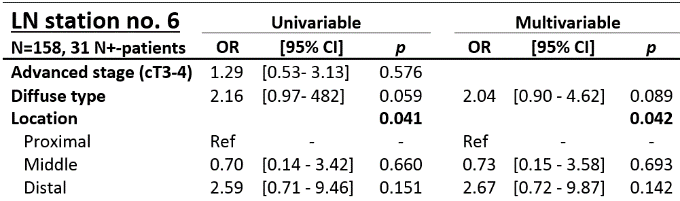

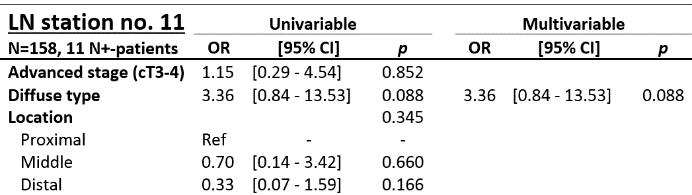

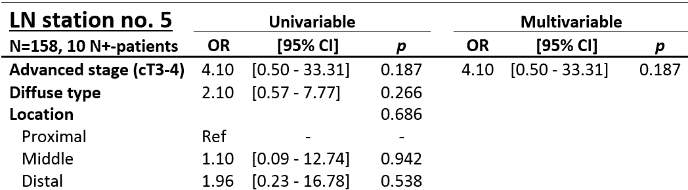

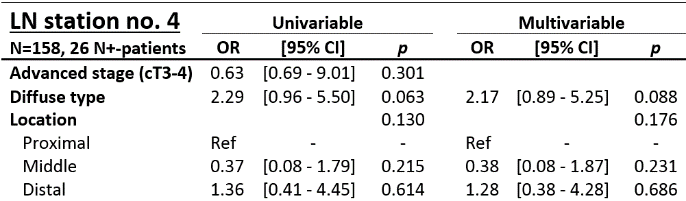

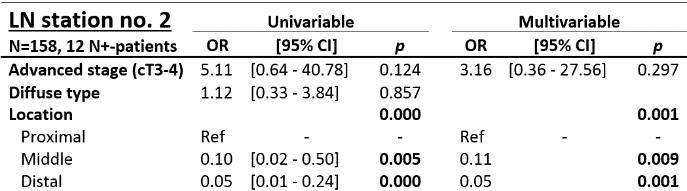

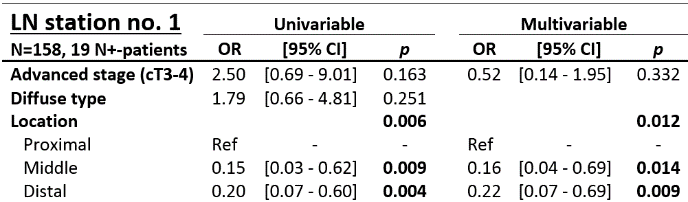
***Supplementary Table 2.* Predictors of LN metastases (N0 versus N+) for only NAC-treated patients (n=158), for each LN station separately.**


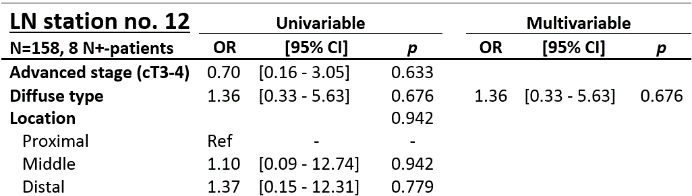


LN = lymph node. NAC = neoadjuvant chemotherapy. OR = odds ratio. 95% CI = 95% confidence interval.

***Supplementary Table 3.* Incidence of skip-metastases in lymph node stations.**

| **Skip-metastases** | **N = 212 (***100%*) |
| --- | --- |
| **Skip metastases present** | 14 (7) |
| **Lymph node stations involved**  No. 7  No. 8  No. 9  No. 11  No. 12 | 7 (3)  8 (4)  5 (2)  3 (1)  1 (0.5) |
| **Number of skip-stations involved**  1 station  2 stations  3 stations | 8 (4)  2 (1)  4 (2) |
| **Disease stage**  Early stage (cT2N0)  Advanced stage (cT3–4 and/or cN+) | 1 (0.5)  13 (6) |
| **Tumor location**  Proximal  Middle  Distal | 1 (0.5)  4 (2)  9 (4) |

Skip-metastases do not involve the perigastric nodal stations (no. 1–6), but metastasize only to remote stations (no. 7–9, 11 and 12a).

***Supplementary Table 4.* Response to neoadjuvant chemotherapy (NAC) in the primary tumor and lymph nodes for intestinal versus diffuse tumors.**

| **Patients treated with NAC N=158** | **Intestinal**  **N=88** | **Diffuse**  **N=67** | ***p-value*** | **Missing** |
| --- | --- | --- | --- | --- |
| **Mandard-TRG (primary tumor)**  TRG 1  TRG 2  TRG 3  TRG 4  TRG 5 | 9 (*10*)  5 (*6*)  24 (*27*)  28 (*32*)  21 (*24*) | 4 (*6*)  3 (*5*)  23 (*34*)  17 (*25*)  19 (*28*) | 0.678* | 2 (1) |
| **Regression in lymph nodes**  Yes  No | 16 (*18*)  72 (*82*) | 9 (*13*)  57 (*87*) | 0.449^#^ | 1 (*1*) |

NAC = neoadjuvant chemotherapy. TRG = tumor regression grading. Percentages may not add up to 100% due to rounding.

Lauren classification was missing for 3 patients. Regression in lymph nodes was missing for 1 patient with a diffuse tumor.
***** Fisher’s exact test.
**#** *Χ^2^*-test.
